# Systematic Review on the Creep of Fiber-Reinforced Concrete

**DOI:** 10.3390/ma13225098

**Published:** 2020-11-12

**Authors:** Nikola Tošić, Stanislav Aidarov, Albert de la Fuente

**Affiliations:** Civil and Environmental Engineering Department, Universitat Politècnica de Catalunya, Jordi Girona 1–3, 08034 Barcelona, Spain; stanislav.aidarov@upc.edu (S.A.); albert.de.la.fuente@upc.edu (A.d.l.F.)

**Keywords:** steel fiber reinforced concrete, polymeric fiber reinforced concrete, polymeric fiber, steel fiber, beam, crack, deflection, creep, shrinkage, modeling

## Abstract

Fiber-reinforced concrete (FRC) is increasingly used in structural applications owing to its benefits in terms of toughness, durability, ductility, construction cost and time. However, research on the creep behavior of FRC has not kept pace with other areas such as short-term properties. Therefore, this study aims to present a comprehensive and critical review of literature on the creep properties and behavior of FRC with recommendations for future research. A transparent literature search and filtering methodology were used to identify studies regarding creep on the single fiber level, FRC material level, and level of structural behavior of FRC members. Both experimental and theoretical research are analyzed. The results of the review show that, at the single fiber level, pull-out creep should be considered for steel fiber-reinforced concrete, whereas fiber creep can be a governing design parameter in the case of polymeric fiber reinforced concrete subjected to permanent tensile stresses incompatible with the mechanical time-dependent performance of the fiber. On the material level of FRC, a wide variety of test parameters still hinders the formulation of comprehensive constitutive models that allow proper consideration of the creep in the design of FRC elements. Although significant research remains to be carried out, the experience gained so far confirms that both steel and polymeric fibers can be used as concrete reinforcement provided certain limitations in terms of structural applications are imposed. Finally, by providing recommendations for future research, this study aims to contribute to code development and industry uptake of structural FRC applications.

## 1. Introduction

In recent decades, one of the most promising types of concrete, for both structural and non-structural applications has become fiber-reinforced concrete (FRC), i.e., concrete produced with steel or polymeric fibers which can bring tangible technical and economic benefits [1]. Studies using multi-criteria decision making methods, considering social, economic and environmental sustainability, have also shown that FRC can be more sustainable than reinforced concrete (RC) for different infrastructure applications, for which the use of fibers is technically viable (as unique reinforcement or in combination with traditional steel rebars) [2,3,4]. Considering the immense quantities of concrete produced globally, i.e., more than 25 billion tons annually [5], such advances towards more sustainable solutions are crucial.

In developed countries, more than 50% of total concrete produced is used in structural applications [6]. Therefore, FRC has been tested increasingly as a solution for partially or even completely replacing reinforcement for applications such as ground-supported slabs [7,8,9], pavements [10,11], roads, tunnel linings [12,13,14,15,16,17,18], pipe sewer lines [19,20], and flat slabs [21,22,23,24,25], which means that it is increasingly used in elements exposed to bending, resisting gravitational, long-term loads. Owing to extensive research over past years, structural design of FRC has been incorporated into several design codes, such as the *fib* Model Code 2010, ACI 318, Italian and the Spanish Code [26]. Nonetheless, research has so far focused mostly on short-term material and structural properties and this has left some FRC design aspects still in the early stages of research. One such aspect is the time-dependent behavior, and particularly, creep of FRC.

Even without considering the effects of fibers, the quantification of the mechanical behavior of RC structures is complex due to effects such as shrinkage, creep, and cracking [27]. FRC introduces further complexities, especially considering that FRC itself may be produced with different types of fiber such as steel fibers (steel fiber-reinforced concrete, SFRC) or polymeric fibers (polymeric fiber reinforced concrete, PFRC) that can exhibit different mechanical properties. In general, among the aspects to be considered, is the progressive creep or damage of the fiber-matrix interface and the debonding and pull-out of the fibers [26] as well as the susceptibility of fiber filaments to tensile creep, in the case of certain polymeric fibers [28] when subjected to stress levels incompatible with material properties.

When these effects are combined with creep and shrinkage of the concrete, FRC members may be subjected to increasing crack widths and, consequently, potential loss of serviceability, durability and, eventually, mechanical performance. In this regard, crack width is a governing parameter in steel-based reinforcements for concrete and extensive experimental programs have found that chloride-induced corrosion [29,30] and embrittlement [31] mechanisms are crack width sensitive. These phenomena are unexpected when polymer-based materials are used as concrete reinforcements. However, polymeric fiber creep in cracked-sections may lead to loss of mechanical capacity if these are subjected to permanent tensile stresses of magnitudes incompatible with the time-dependent mechanical properties of the fibers, tensile creep of cracked-sections also being a crack width-sensitive phenomenon. This drawback can be solved through the use of hybrid solutions (fibers and steel reinforcing bars, “hybrid-FRC”) [26].

Thus, it is evident that creep of cracked FRC elements has clear design and structural implications and should be carefully considered. Although there exist some general provisions and recommendations (rather limitative and restrictive due to the lack of consistent research), it is still necessary to derive reliable design and calculation methods for serviceability limit state (SLS) analysis of cracked FRC structures subjected to long-term bending.

While previous studies have focused only on a single fiber type or single level (fiber, material, structure), a systematic review of existing literature on the response of FRC and hybrid-FRC subjected to creep is necessary from a comprehensive and bottom-up perspective. For this purpose, this paper presents a critical assessment of published studies collected through a methodical literature search. Hence, the aim of the study is to provide insight into the main influencing parameters of FRC creep, methods for experimental characterization as well as theoretical formulation of FRC time-dependent behavior. In Section 2, details about the literature search are presented in terms of databases used, search terms and justification of publication screening. In Section 3, research on the material level is presented, from studies on the creep behavior of individual fibers and fiber material (steel and polymeric macrofibers) to long-term studies on the level of concrete specimens. In Section 4, existing research on the structural level is presented (full-scale tests on FRC and hybrid-FRC elements under long-term bending) as well as existing analytical SLS design proposals. Each section ends with a synthesis of the presented knowledge, a critical assessment of literature, and guidelines for future research. Finally, Section 5 summarizes the entire study with a concise overview and recommendations for future work that can lead to a consistent and comprehensive SLS design methodology for FRC structures.

## 2. Methodology

The first step in the study was a systematic literature search. For this purpose, journal and conference articles dealing with the creep behavior of SFRC and PFRC, on material and structural levels, were considered. For the literature search, the online databases of Scopus [32] and Web of Science [33] were used, complemented with personal archives compiled previously.

The following search terms were used for Article Titles in Scopus and Web of Science:

1. “fib** reinforced concrete” AND “long-term”
2. “fib** reinforced concrete” AND “time-dependent”
3. “fib** reinforced concrete” AND “creep”
4. “synthetic” AND “fib**” AND “creep”
5. “polypropylene” AND “fib**” AND “creep”

The syntax “fib**” was used to cover both British and American English spelling (fibre vs. fiber). Search terms 1–3 were used to find research at the concrete specimen and structural member level, whereas search terms 4–5 were used to find on research on the single fiber or fiber material level (focusing on “synthetic/polymeric” fibers as these are more prone to undergo creep and polypropylene fibers as the most widely used among this type of fiber).

The initial search yielded a total of 250 studies: 125 on Scopus, 83 on Web of Science and 42 from the personal archive of the researchers. The next step was the removal of duplicate studies which led to the removal of 90 studies, leaving a total of 160 distinct journal and conference articles.

The remaining articles were screened for language (only studies in English were retained) and topic (only studies on concrete and time-dependent behavior were retained—studies on, e.g., asphalts, fiber composites and durability were excluded). This led to the removal of 69 studies, leaving 91 studies in the database.

Finally, through the institutional access available to the researchers, 19 studies could not be accessed. Therefore, the final number of studies considered in the review was 72. These studies were divided into three groups: (1) fiber level, (2) concrete level, and (3) structural level. When dividing the studies according to their content, certain studies were categorized into more than one group. Therefore, the 70 studies were divided into (overlapping) groups of 10, 43, and 23 studies for groups (1), (2), and (3), respectively, summarized in Table 1.

Any systematic review introduces a certain bias. Herein, the bias consists in several factors. First, the selection of studies only in English potentially excludes a body of knowledge in other languages. However, considering that the large majority of studies indexed in Scopus and Web of Science are in English, this bias is considered not significant. Second, only journal and conference articles are considered, and documents such as reports and theses are excluded. This bias can also be considered negligible as the number of these documents is not large, and, e.g., doctoral theses are typically accompanied by journal publications of the same content. Finally, a certain bias is introduced by the databases themselves; however, as the most renowned and accepted databases, Scopus and Web of Science were considered appropriate.

## 3. Research on the Material Level

### 3.1. Creep Behavior of Fibers and Fiber–Concrete Bond

As a multi-level phenomenon, the creep behavior of FRC is also influenced by the behavior of the fibers themselves as well as their bond with the concrete matrix. Gettu et al. [28] identify creep of individual fibers or filaments and the progressive creep of the fiber–concrete interface as significant factors that can lead to crack width increase, as well as durability and serviceability issues in cracked FRC elements under sustained loading. However, a fundamental difference exists between steel fibers and polymeric fibers. In the case of PFRC, the creep of individual fibers is the dominant effect in PFRC; whereas in SFRC, the pull-out creep becomes the dominant phenomenon at the material level, as steel fibers do not undergo any considerable creep at normal temperatures [28].

#### 3.1.1. Creep Behavior of Individual Fibers or Filaments

Unlike steel fibers, which undergo negligible creep at room temperature, polymeric fibers can experience creep, particularly at elevated temperatures. This behavior is caused by their viscoelasticity; particularly, more crystalline and cross-linked polymers are less susceptible to creep [28]. In cracked FRC sections, the creep of polymeric fibers, most notably polypropylene, will lead to an increase of crack width which will in turn cause relaxation in the fibers leading to a decreased bridging effect and further crack width increases [28].

Studies on the creep behavior of polypropylene fibers date back to the 1960s and the works of Hadley and Ward [40]. These authors were among the first to perform creep and recovery tests on polypropylene filaments and propose mathematical formulations for the entire range of behaviors exhibited by polypropylene [40]. Hadley and Ward [40] found that the relationship between creep compliance (function describing the relation between total strain and stress) and stress for six fiber monofilaments—produced in different ways such as drawing and spinning—generally consists of four regions assessed qualitatively: (1) a region at low stresses in which specific creep (creep strain divided by stress) is independent of stress and linear viscoelastic behavior is observed, (2) a region at higher stresses in which specific creep develops parabolically relative to stress; this is followed by (3) a region in which specific creep is linearly related to stress, and finally, (4) a region at very high stresses, in which specific creep is once more independent of stress. Similar behavior was confirmed also by other authors [41].

**Figure 1:**
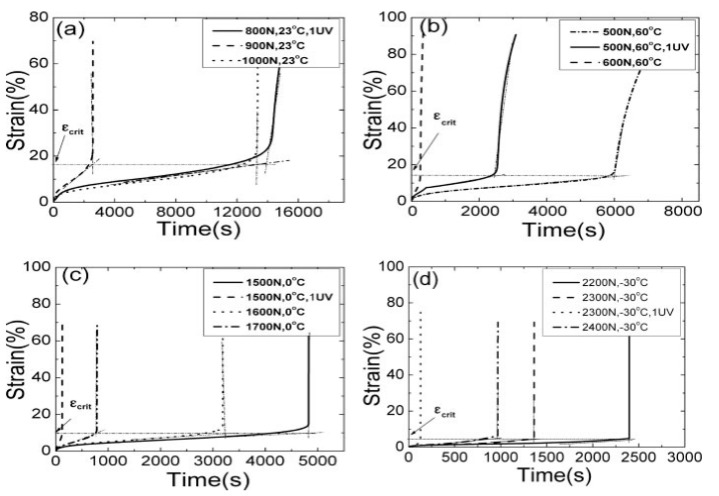
Values of *ε*_crit_ for polypropylene sample PP1 under different aging conditions (authorized reprint from [34]): effect of varying stress and UV intensity under (**a**) 23 °C, (**b**) 60 °C, (**c**) 0 °C and (**d**) −30 °C.

Already by 1980, Takaku [42] had studied creep failure of isotactic polypropylene fibers and found it to be highly dependent on temperature (temperatures between 40 °C and 130 °C were used). More recently, Liu et al. [34] studied the creep behavior of four types of polypropylene under combined effects of temperature, ultraviolet light, and tensile stress. The authors of study [34] tested polypropylene formed into dumbbell-shaped tensile bars 150 × 10 × 4 mm—this is an important limitation to extrapolating the results of this study to polypropylene fibers used in FRC which are produced by a stretching process. The creep tests were performed under a stress lower than the polypropylene yield strength (27–36 MPa for the polypropylene tested in the study). Different temperatures were used (−30 °C to 70 °C), different loads (500–2400 N) and different ultraviolet (UV) light intensities applied for 12 h (300 and 600 μW/cm^2^); and the time to failure was measured. As for the variables chosen in this study, it should be noted that when using fibers in FRC, due to the process of their addition directly in the concrete mix and subsequent embedding in the matrix, ultraviolet radiation is expected to have a negligible effect. Liu et al. [34] found that changes in temperature had the most significant effect on the creep rate of polypropylene. As an example, for sample PP1 under a load of 500 N, at 50 °C there was no creep failure; at 60 °C creep failure occurred after 18,000 s and at 70 °C after 1800 s. The effect of temperature was explained by the increase of the free volume of polypropylene and the subsequent weakening of intermolecular forces. At the same time, under a constant temperature, increasing load led to an increase of the creep rate. For example, when sample PP1 was at a temperature of 23 °C and a load of 800 N, no creep failure occurred; however, increasing the load to 900 N led to creep failure after 13,000 s and increasing the load to 1000 N led to creep failure after 2000 s. The effect of stress was explained by the decrease of the barrier for bond dissociation that enables the movement of molecular chains. The most important finding by the authors of study [34] is that of a critical failure strain, *ε*_crit_*,* that varied depending on temperature but was not affected by tensile stress or ultraviolet light irradiation [34], Figure 1. At room temperature (23 °C), *ε*_crit_ varied between 12% and 17% for the different polypropylene types and increased/decreased with increasing/decreasing temperature.

Fouda et al. [39] tested the influence of temperature on creep deformation of polypropylene fibers based on optical measurements (interferometry). Undrawn polypropylene fibers were subjected to a constant load for up to 230 min under temperatures of 23 °C, 30 °C, and 40 °C. These authors found that the creep rate (increment of creep over an increment of time) firstly decreased with increasing strain (primary creep) and then remained constant (secondary creep). Nonetheless, up to 5 min, the creep behavior seemed to be independent of temperature. Fouda et al. [39] were able to successfully match the observed behavior to a model consisting of a series connection of a two-component Kelvin chain:

(1) $$J\left(t\right)=\frac{1}{E_{1}}\cdot \left(1-e^{-t/\tau_{1}}\right)+\frac{1}{E_{2}}\cdot \left(1-e^{-t/\tau_{2}}\right)$$

where *J*(*t*) is the compliance function at time *t*, *E*_1_ and *E*_2_ are the spring moduli, and *τ*_1_ and *τ*_2_ are the retardation times of the Kelvin models 1 and 2, respectively. Finally, these authors also found that fiber yield stress and modulus of elasticity decreased with increasing temperature, whereas the fiber yield strain increased with increasing temperature [39]. It should be noted for this study as well that undrawn fibers were used, i.e., fibers not produced in the same way as those intended for application in FRC. Therefore, a potentially different behavior of drawn polypropylene fibers could be expected, thus warranting further investigations into this topic.

Vrijdaghs et al. [35] tested two types of polypropylene macrofibers for uniaxial tensile creep. A total of 26 samples (14 of fiber A and 12 of fiber B) were tested at a temperature of 20 °C and relative humidity (RH) of 60%. Five different load levels were considered, from 22% to 63% of the fiber strength. The time to failure and strain at failure were recorded. The authors [35] found that all samples underwent failure in the secondary creep phase. The time to failure ranged from several hours for samples loaded to 63% of fiber strength to several months for fibers loaded to 22% of their strength. The strains at failure were significant (40–100%) and the creep coefficients (ratios of creep strain to instantaneous strain) were generally larger than 10. However, these results only describe the behavior of polypropylene alone, i.e., the observed behavior is not expected to be replicable at the structural level once fiber–matrix interaction comes into play as well as the presence of steel reinforcement in structural members.

#### 3.1.2. Pull-Out Behavior of Fibers

**Figure 2:**
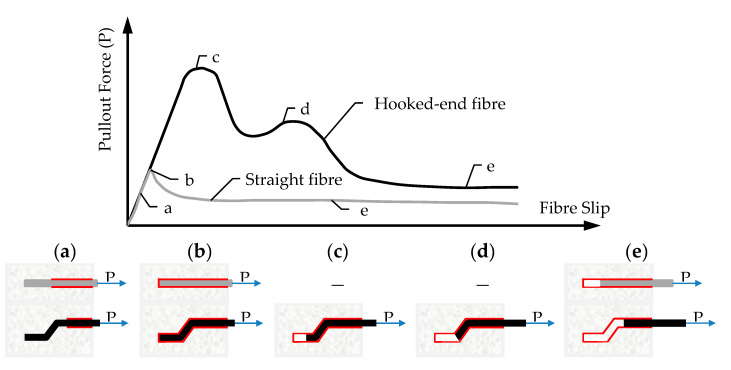
Example of debonding and fiber pull-out of straight and hooked-ended fibers (adapted from [28]). (**a**) Partial debonding, (**b**) Full debonding, (**c**) Plastic deformation of fiber hook (1), (**d**) Plastic deformation of fiber hook (2) and (**e**) pull-out.

Whereas in the case of polymeric fiber reinforced concrete (PFRC) temperature has an effect mostly through single fiber or filament creep, in the case of SFRC the effect of temperature is mostly exerted through changes in the fiber–concrete bond [28]. This process consists of debonding and frictional pull-out, as shown in Figure 2. As for PFRC, particularly polypropylene fiber reinforced concrete (PPFRC), the Poisson’s ratio (*ν*) of polypropylene (0.40–0.45) causes significant lateral contraction of the fibers facilitating debonding [28].

Vrijdaghs et al. [36] performed creep pull-out tests on two types of polypropylene macrofibers for which fiber creep was assessed previously [35]. These authors used a test setup in which a single fiber was embedded over varying lengths (10–30 mm) in a concrete cylinder while the other end of the fiber was clamped between steel plates over a length of 65 mm with 20 mm of free length. Then, the fibers were tested under a temperature of 20 °C and relative humidity (RH) of 60% under loads corresponding to 25–75% of the pull-out strength obtained in short-term tests (*P*_max_). It was found that all specimens loaded above 40% of *P*_max_ failed within 60 days due to complete fiber pull-out. The authors of study [36] calculated the initial debonded length of the fibers of *l*_db_ = 14.4 mm consisting of 3–4 mm within the concrete (4–5 times the fiber diameter) and ~10 mm in the clamps. Over this length, the creep of the fiber itself was considered [35]. Once these results were superimposed on the long-term pull-out results, it was found that there is an excess of creep deformation above the single fiber creep. The authors concluded that this was due to the progressive debonding caused by time-dependent Poisson contraction of the fiber [36].

**Figure 3:**
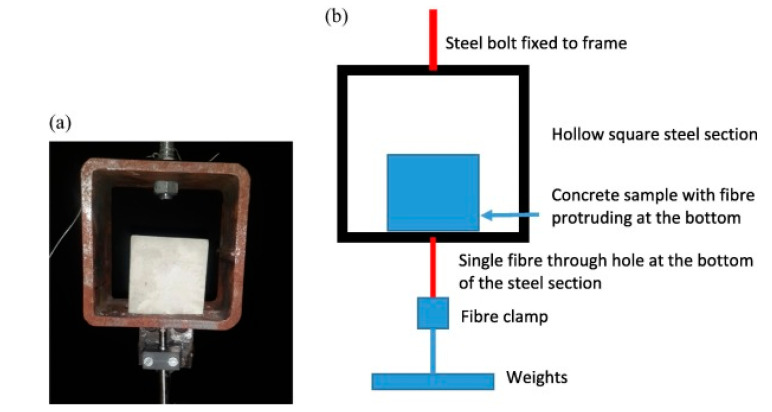
(**a**) Sample placed in position on steel section for the pull-out creep test (**b**) diagram of the setup [38].

Pull-out creep tests on PFRC specimens were also performed by Babafemi et al. [38] who tested three types of polymeric macrofibers embedded in 50 mm cubes and loaded to 50% of the pull-out strength for 30 days, Figure 3. These authors found a significant effect of fiber type with one fiber failing after 22 days, whereas the pull-out displacement of the other two fiber types gradually decreased. X-ray computed tomography images of the samples were taken and it was found that immediately after loading, an initial debonding occurs and the instantaneous axial displacement is actually a consequence of the elongation of the fiber over the debonded length, and not of pull-out. Over time, the dominant effect becomes fiber creep over the debonded length which leads to Poisson contraction, loss of friction and subsequent pull-out.

A similar study to that of Vrijdaghs et al. [36] was performed by Nieuwoudt et al. [37], but on SFRC using hooked-end steel fibers. Among other properties, the authors performed single fiber pull-out creep tests on single steel fibers embedded in 100 mm cubes. In these tests, the variables were load level, fiber orientation angle, fiber mechanical anchorage, and fiber pre-slipping. The tests were performed for 240–250 days. It was found that increasing the load level led to increasing pull-out. The results for different fiber orientation angles did not show a clear trend. As for mechanical anchorage, the more kinks the steel fiber had, the higher was the pull-out creep—this was ostensibly due to the fact that specimens with steel fibers with more kinks were actually loaded to higher absolute loads, and thus, caused higher creep, as well as the fact that more concrete is in the zone around the kinks, exposed to localized sustained compressive loading leading to higher creep.

#### 3.1.3. Summary of the Results on the Creep Behavior of Fibers and Fiber–Concrete Bond

The studies that were analyzed reveal important conclusions at the material level. In terms of individual fiber creep—beside the fact that it is negligible for steel fibers—the creep of polymeric, particularly polypropylene fibers needs to be taken into account under certain circumstances. All studies point to a clear influence of temperature on polypropylene creep behavior, as well as the existence of a critical failure strain that is temperature-dependent, but load-independent [34]. Furthermore, it is important that studies have confirmed the applicability of Kelvin chain models for describing polypropylene creep behavior [39]. What remains to be formulated is a temperature dependency of the series Kelvin chain model.

At the level of fiber–concrete bond, several pull-out tests with important conclusions were presented. In the case of SFRC, fiber shape (number of kinks in hooked-end fibers) was shown to cause an important effect through complex stress localizations around the kinks leading to potentially higher pull-out creep [37]. In PFRC, the creep of individual fibers needs to be superimposed on the pull-out creep that seems to be of secondary importance in this case [38]. A potential way forward in this area is the proposal of time-dependent and temperature dependent Kelvin chain models that would consider only pull-out creep in the case of SFRC and combined effects of individual fiber creep and pull-out creep in the case of PFRC.

### 3.2. Creep Behaviour of Fiber-Reinforced Concrete (FRC)

So far, the largest number of research studies on the creep behavior of FRC was performed on the material level of specimens in long-term compression, tension, and bending. However, these tests are time-consuming and with a large number of influencing factors, this challenging proper execution. Even though the number of performed tests is significant, almost all of them have been performed with different parameters, from the type of FRC, to the type of test, applied load, duration, or type of measurements recorded. This poses significant challenges in interpreting the results, synthesizing conclusions, and proposing constitutive models.

**Figure 4:**
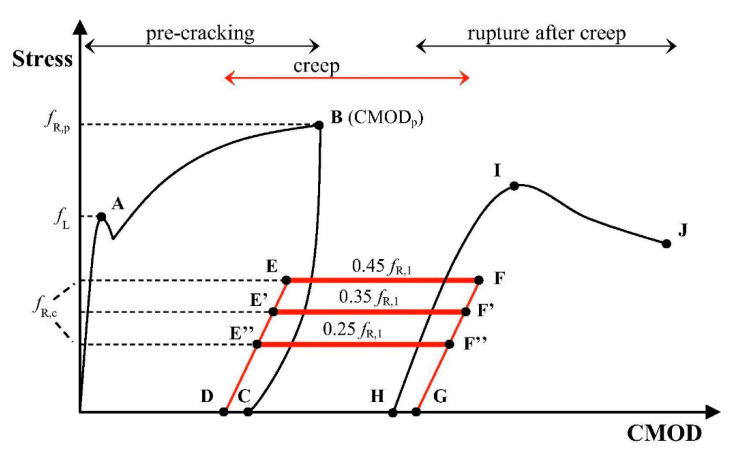
Main stages of the cracked fiber-reinforced concrete (FRC) creep test methodology [56].

In terms of tensile creep of FRC—which is of primary interest in this paper—a typical test procedure is shown in Figure 4. Whether the test is uniaxial tension or bending, the specimen is first pre-cracked to a pre-defined crack-opening (point B) for which the residual strength *f*_R_ is determined. This pre-crack width is usually within the range of 0.2–0.5 mm but this can vary significantly. Further, the average crack opening is mostly used as a quantification of the crack opening [75]. Subsequently, the specimen is unloaded (B-C) and moved to the long-term testing frame. This is important to note, since the specimen typically is tested in different machines for short-term and long-term characterization. This can entail a change in boundary conditions and undesired influences [75].

As pre-cracking is normally performed at low crack opening rates, creep deformations develop during this stage as well. Part of this deformation is recovered in the unloaded state (C-D). Afterwards, the specimen is reloaded to a fraction of the residual stress measured during pre-cracking (D-E/E’/E’’), *σ*_c_ = *α*·*f*_R_, where *α* is the so-called “creep load ratio,” typically chosen in the range of 30–70%; nonetheless, its proper justification is very important. The reloading time *t*_L_ should be as short as possible, in order to limit the interference of instantaneous and delayed deformations [75]. During the long-term tests, deformations increase at a constant load (E-F/E’-F’/E’’-F’’). At the end of the long-term test (if failure did not occur), the specimen is unloaded (F/F’/F’’-G) and part of the long-term deformation is recovered (G-H). Finally, the specimen might be reloaded to failure in a final short term test (H-I-J).

In the following subsections, studies on compressive creep and shrinkage of FRC and tensile creep in uniaxial tension and bending are summarized and existing analytical and numerical models are presented with an identification of existing knowledge gaps and recommendations for future research.

#### 3.2.1. Effect of Fibers on Compressive Creep and Shrinkage of FRC

Although shrinkage and compressive creep are important phenomena, they have been often overlooked in research on FRC. The benefits of fiber reinforcement in terms of reducing plastic shrinkage are well-known and acknowledged [104] and can be significant—for example, Pešić et al. [54] report 70–80% of plastic shrinkage reduction with moderate amounts of steel and plastic fibers (0.40–1.25%). However, tests on drying shrinkage and creep are less numerous.

The ACI Committee 544 report on FRC [105] suggests that the addition of less than 1% of steel fibers (80 kg/m^3^) does not induce an effect on compressive creep. Nonetheless, the results of individual researchers can differ. For example, Nakov et al. [52] tested C30/37 concretes with 0, 30, and 60 kg/m^3^ of steel fibers in compression for 400 days, exposed to a compressive stress of 7.5 MPa (stress-to-strength ratio of approximately 0.2). These authors found creep to decrease with increasing steel fiber content. After fitting the analytical B3 creep prediction model [106] to their experimental results and extrapolating to 100 years, the creep coefficient was reduced by 11.1% and 17.8% for SFRC with 30 and 60 kg/m^3^ of steel fibers, respectively, relative to the ordinary Portland cement concrete (OPC). Similarly, Chern and Young [62] also found that the inclusion of up to 2% of steel fibers reduces both creep and shrinkage with increasing fiber volume *V*_f_. Additionally, Chern and Young [62] noted that the effect of fibers increased over time, i.e., the differences between SFRC and OPC increased over time due to more activation of fibers as the concrete underwent creep. In a study on high-performance FRC, Afroughsabet and Teng [58] tested FRC with only steel fibers and with a mix of two different steel fibers and a mix of two different steel fibers and polyvinyl alcohol fibers. These authors found that the addition of fibers decreased creep and shrinkage and that adding mixes of fibers was beneficial to the reduction of deformation.

Contrary to these results, Błyszko [60] tested SFRC and OPC and found a significant increase in compressive creep when adding steel fibers. However, it should be noted that the test lasted only around 15 days and the concretes were exposed to high compressive stresses equal to 40% and 85% of their compressive strength. In this case, even the 40% load level can be considered to fall under non-linear creep conditions where significant microcracking is present in the concrete, this potentially affecting the fiber bond and causing damage to the matrix. Since such high compressive stresses are unlikely to occur under typical service conditions of FRC structural applications, they can be considered of less significance for design implications.

Overall, the question of FRC compressive creep and shrinkage is empirical and its resolution depends on significantly more tests being performed. Such results could then be included in existing creep and shrinkage databases, such as the NU-ITI [107], and existing models such as the *fib* Model Code 2010 [108] and B4 [109] could be adapted for FRC. Until then, considering expected stress levels in FRC structural applications and typical fiber contents, effects of fibers on creep and shrinkage could be disregarded.

#### 3.2.2. Long-Term Uniaxial Tension Tests

**Figure 5:**
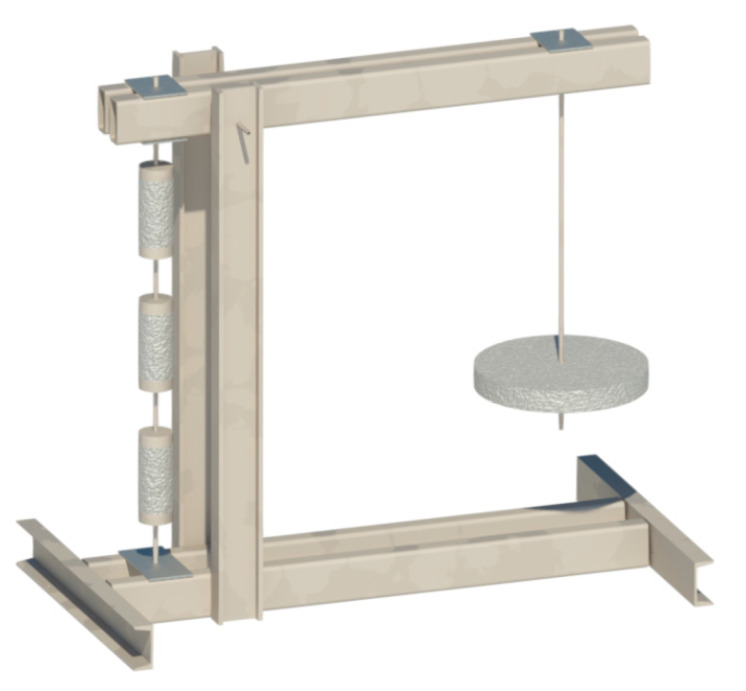
Experimental setup for long-term uniaxial tension tests (adapted from [75]).

Theoretically, long-term uniaxial tension tests should be a preferable choice for testing the tensile creep of FRC. However, performing them carries significant challenges in equipment and measurement design such as the requirement for relatively large samples and the possible occurrence of secondary moments causing stress redistribution [75]. Therefore, such studies are less numerous than long-term bending tests and practical experience is quite limited. A typical long-term uniaxial tension test setup is shown in Figure 5.

An overview of the setup and parameters of long-term uniaxial tension tests is provided in Table 2. Two of the studies tested SFRC with hooked-end steel fibers and two studies tested PPFRC. The fiber volume is relatively uniform as are specimen type, climate conditions, test duration, and fiber aspect ratio. There is a somewhat larger variation in the pre-crack width selected for the test with an average close to 0.4–0.5 mm. Finally, load level was also widely varied in the test. Notably, all studies except that by Zhao et al. [49] defined the load level as a percentage of the residual strength *f*_R_ at the selected pre-crack width. However, Zhao et al. [49] defined it as the percentage of maximum pre-cracking load *P*_max_. Zhao et al. [49] and Nieuwoudt et al. [37] explicitly state that shrinkage was also measured and taken into account in the analysis.

A summary of the results is shown in Table 3 where *f*_max_ is the maximum pre-cracking stress, *f*_R1_ and *f*_R3_ are residual strengths at crack widths of 0.5 and 2.5 mm, respectively, and *ϕ*_w_ is the crack width creep coefficient defined as the ratio of the increase in crack width over time *w*_creep_ divided by the initial crack width after loading in the creep test *w*_init_. However, it should be noted that at time *t* in the long-term test, the total crack width *w*_tot_ is composed of the following:

(2) $$w_{\mathrm{tot}}=w_{\mathrm{irr}}+w_{\mathrm{inst}}+w_{\mathrm{creep}}+\delta_{sh}$$

(3) $$\varphi_{w}=w_{\mathrm{creep}}/w_{\mathrm{inst}}$$

where *w*_irr_ is the irrecoverable crack opening upon unloading in the short-term tests and *δ*_sh_ is the shrinkage of the specimen.

The results in Table 3 reveal several interesting outcomes. Firstly, in the case of SFRC, the crack width creep coefficient is similar to the concrete creep coefficient in tension. Since in the case of SFRC there is no creep of the fibers, the only creep in tension comes from the fiber-concrete bond. This is actually a complex superposition of compressive and tensile concrete creep. Since tensile creep of concrete is in the same order of magnitude as compressive creep, with studies claiming it has either similar [110] or 50–100% greater values [111], this explanation seems plausible.

However, for PPFRC the situation is quite different. For the study by Babafemi and Boshoff [79] *ϕ*_w_ values could not be extracted. The authors of that study note that there was no crack width stabilization even for specimens loaded to 30% of pre-cracking residual strength, whereas specimens loaded to 60% and 70% failed within 10 and 1 days, respectively. In the case of Vrijdaghs et al. [50], specimens loaded to 30% exhibited very low crack width increases that did not surpass initial crack width values even after 180 days. However, when loaded to 45% of the residual strength, very large *ϕ*_w_ values were recorded and initial crack widths were surpassed within hours. These results point to the special importance of controlling for individual fiber creep in the case of PFRC.

#### 3.2.3. Long-Term Bending Tests

**Figure 6:**
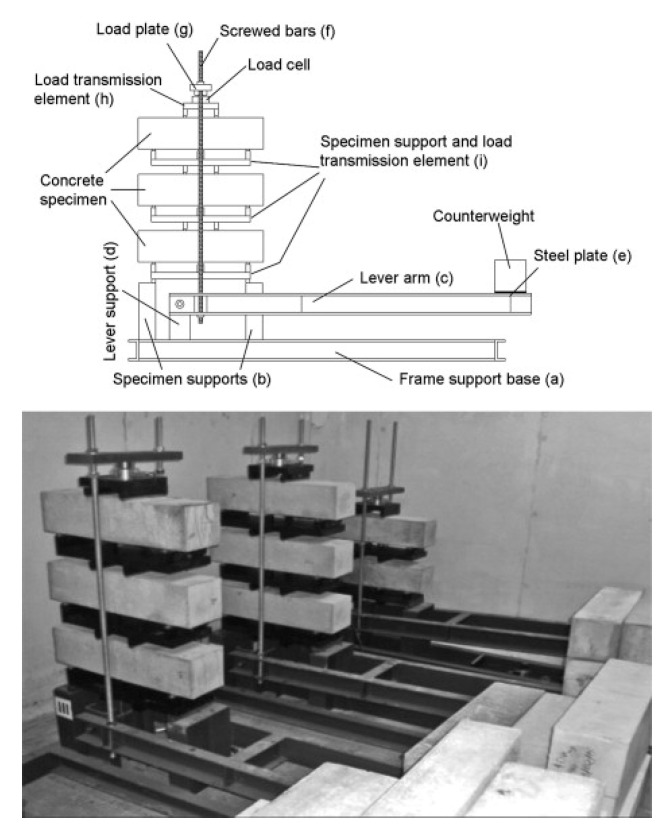
Experimental setup for long-term bending tests [55].

The majority of long-term tests on FRC on the material level were performed in the form of long-term bending tests on prismatic specimens. Such tests typically employ a lever system in order to maintain a constant load, and generally, several stacked specimens are used, Figure 6. The use of stacked specimens means that not all of them are exposed to the same load; however, considering the scatter of FRC residual strength, this can typically be overcome by careful arrangement of the specimens [75]. In the vast majority of cases, four-point bending is used; however, this means that the long-term test configuration differs from the typical short-term characterization (e.g., using the EN 14,651 three-point bending test [112]).

Unlike uniaxial tension tests in which the crack width, i.e., crack opening displacement (COD), is directly measured, in bending tests, the measured deformation is usually the crack mouth opening displacement (CMOD), crack tip opening displacement (CTOD) or the mid-span deflection. The results are then reported in terms of creep coefficient representing the ratio of the creep and initial deformation components. It should be kept in mind that the measured deformation is affected by the tensile creep of fibers and the fiber–concrete bond as well as the compressive creep of concrete in the compressed zone and shrinkage. Since the crack can propagate significantly in the cracked section (e.g., up to 100 mm in a 150 mm high cross section [28]), the compressive stress in the cracked zone can become high and cause nonlinear creep and microcracking. Hence, the use of “creep coefficients” obtained directly from bending tests without compensating for compressive creep and shrinkage should be done with caution [28].

An overview of the setup and parameters of long-term bending tests is provided in Table 4. The table reveals that there is a wide variety of parameter choices in the majority of the tests performed so far. Importantly, SFRC and PFRC are relatively equally represented with several studies performing comparative research on their long-term behavior. Furthermore, the majority of the tests are four-point bending tests, although Zerbino et al. [78] demonstrated that the use of three-point bending in long-term tests would not significantly alter the nature of the obtained results. As in uniaxial bending tests, the creep load is selected as a percentage (typically 50%) of the residual strength at the selected pre-crack width (typically 0.5 mm); nonetheless, both the pre-crack widths and load levels can vary significantly. It is important to keep in mind that, from the point of view of SLS design, even the pre-cracking widths of 0.5 mm exceed typically allowed crack widths: for example, the Eurocode 2 crack width limit for RC members under the quasi-permanent combination is 0.3 mm for the majority of ambient exposure classes [113].

As for the results, comparing and analyzing them quantitatively is more difficult than in the case of uniaxial tension tests. Namely, creep coefficients are sometimes reported in terms of CMOD, sometimes in terms of deflections. Additionally, the creep coefficient is sometimes defined based on the initial crack width after loading in the creep test (i.e., compensating for the irrecoverable crack width *w*_irr_) and other times in terms of the initial crack width related to the origin (*w*_irr_ + *w*_inst_) (Figure 4). Finally, shrinkage is not always taken into account, whereas the effect of compressive creep is almost never discussed. Therefore, only a qualitative discussion of the results is meaningful. Generally, as in the case of uniaxial tension tests, the creep of SFRC is not drastic, specimens never experience tertiary creep or failure and creep coefficients are commensurable to those in compression [56,57,71,80]. As for results on PFRC, as in earlier described studies, the deformations tended to strongly depend on temperature. For example, Buratti and Mazzotti [83] tested PFRC under increasing temperature from 20 °C to 50 °C which had a major effect on their behavior, increasing deformation and even leading to failure. Kurtz and Balaguru [47] compared polypropylene and nylon FRC pre-cracked to 0.75 mm. The authors of study [47] found that the “maximum infinitely sustainable stress” (i.e., stress that did not lead to creep failure) was 24.9% for PPFRC and 38.3% for nylon FRC. Nonetheless, as stated above, the generally adopted pre-crack widths in these studies exceed common code limitations [113] and, therefore, the “maximum infinitely sustainable stresses” are most likely higher at crack widths corresponding to code limits (0.2–0.4 mm), probably approaching 60% of residual strength at those crack widths. In terms of other parameters, Zerbino et al. [53] found that varying beam width did not significantly affect results, at least for SFRC, but increasing width did reduce variability. Zerbino et al. [53] also did not find any effect of creep deformation on ultimate strength.

One parameter that was identified as potentially having explanatory power was the crack opening rate *COR* [56,71,78]:

(4) $$COR^{i-j}=\left(CMOD_{\mathrm{ct}}^{j}-CMOD_{\mathrm{ct}}^{i}\right)/\left(t_{j}-t_{i}\right)$$

where *COR^i^*^–*j*^ is the crack opening rate in time increment *i*–*j*, *CMOD_ct_^i^* and *CMOD_ct_^j^* are total crack opening at times *t*_i_ and *t*_j_, respectively. *COR* tends to stabilize after a few weeks; hence, measurements should be performed for at least 90 days [71]. However, much more work is needed in this direction in order to define conformance and acceptance criteria in terms of *COR*.

Nonetheless, the variability of parameters in tests and the variability of FRC long-term properties have made it difficult to identify clear influences of certain parameters. For example, Llano-Torre et al. [68] performed a quantitative analysis of literature on long-term uniaxial and bending tests of SFRC, PFRC and glass fiber FRC. These authors analyzed two crack width creep coefficients at 90 days: one related to the origin (taking into account *w*_irr_ + *w*_init_), *ϕ*_o_ and another taking into account only *w*_init_ (as the one in Table 3), *ϕ*_c_. Analyzing the relationship between these creep coefficients and applied load level (IFa)—expressed as a percentage of residual strength at pre-cracking (0.5 mm)—a very large scatter was found, obscuring any clear trend and effect of load level (which should be theoretically considered a primary factor).

#### 3.2.4. Modeling the Creep Behavior of FRC

The fact that there is a wide variety of tests, test parameters, and reported results on the long-term behavior of FRC has impeded the proposal of general analytical models. So far, most of the work has been restrained to numerical models of long-term uniaxial tension or bending tests on FRC, typically validated only on a smaller number of experimental results [48,76].

**Figure 7:**
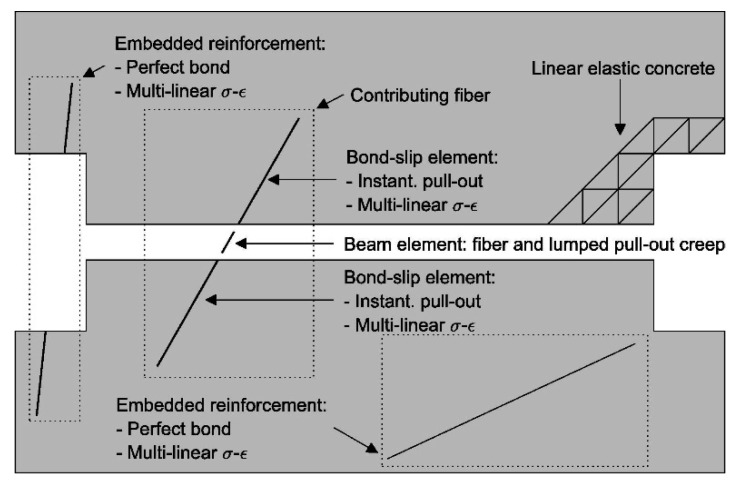
Overview of the finite element model and different element types [76].

Vrijdaghs et al. [76] modeled own uniaxial tension tests on PFRC notched cylinders using a two-phase finite element model. The authors used a MATLAB algorithm for random placing of fibers in a DIANA model [76]. The numerical models consisted of 3D solid elements modeling concrete, embedded reinforcement elements, bond-slip reinforcement elements and beam elements modeling the fibers, Figure 7.

Using this model, Vrijdaghs et al. [76] find a good qualitative description of the experimental behavior, even though instantaneous crack widths are overestimated, probably due to instantaneous load application in the model versus the gradual application in the experiment. Most importantly, these authors found that stresses in the 90% of the fibers crossing the crack were under 10–15% of fiber strength (the specimens were loaded to 30–45% of the residual strength at a pre-crack width of 0.2 mm). The most loaded fibers were under stresses only 20–30% of their strength. Over time, stress redistributed from the most loaded to the less loaded fibers. The simulations were continued up to 50 years, finding no structural failures. The results point to long-term behavior in PFRC being a serviceability limit state problem rather than an ultimate limit state problem [76].

Babafemi and Boshoff [48] also performed numerical modeling in DIANA; however, in this case on long-term bending tests on PFRC. The authors used a mono-phase model, i.e., only concrete, assigning viscoelastic properties only to elements in tension. The long-term behavior of PFRC was modeled using a Kelvin chain of four elements. The authors of study [48] achieved a fair degree of accuracy in modeling time-dependent crack opening. However, it should be noted that concrete compressive creep—with a potentially significant effect in bending tests—was not taken into account.

Considering the results of these two studies, there seems to be a way forward in further investigating viscoelastic constitutive models for numerical analyses of the creep behavior of FRC. However, it remains imperative to include factors such as compressive creep and shrinkage, as well as to find a way to appropriately combine different fiber “positions” in the element (e.g., fully embedded or bridging a crack).

#### 3.2.5. Summary of the Results on the Creep Behavior of FRC

From the analyses of material-level results on long-term testing of SFRC and PFRC specimens it can be seen that the relatively large number of results is yet to be systematically synthesized into analytical models. The large variation in test parameters and reported results is currently one barrier. Furthermore, there is still a lack of fully comprehensive testing that would in one experimental program contain shrinkage tests, compression creep tests and tension tests (uniaxial, bending or both). The results of numerical simulations provide encouraging results that viscoelastic constitutive models so far used for OPC will be applicable to FRC once proper calibration against experimental results is performed.

## 4. Research on the Structural Level

### 4.1. Long-Term Tests on Full-Scale FRC Members

**Figure 8:**
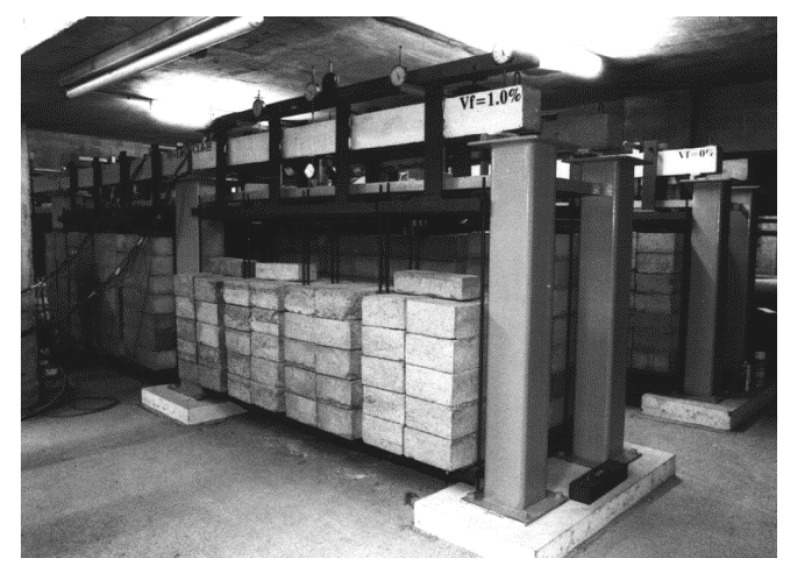
Experimental setup for testing FRC beams (authorized reprint from [98]).

**Figure 9:**
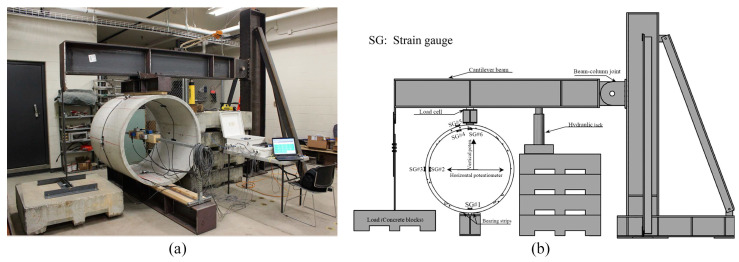
(**a**) Photograph and (**b**) schematic diagram of the long-term test setup [94].

As with other properties of concrete, the long-term behavior of FRC observed on the material level cannot be directly extrapolated to structural behavior. Therefore, full scale long-term tests on FRC members are necessary. However, executing such tests is a challenge due to the large numbers of parameters involved, their time-consuming nature, and significant economic cost. Nonetheless, several researchers have performed such tests on beams and pipes (Figure 8 and Figure 9), with a summary of their main parameters given in Table 5.

While there are not enough results for analyzing in detail the effect of certain parameters, several important general conclusions can be drawn. First, it can be seen that material-level behavior does not translate to structural behavior, particularly in the case of PFRC members without steel reinforcement in the studies by Park et al. [93] and Attiogbe et al. [96]—the tested pipes did not exhibit drastic increases of vertical displacements, nor creep failure, rather, a very minor effect of pre-cracking on the long-term vertical displacements was found (both for buried and unburied pipes). Secondly, in the case of R-SFRC members, the steel fibers always decreased deflections and crack widths. For example, Tan et al. [100] found that after 10 years, deflections of R-SFRC beams with 2% of steel fibers were 36% smaller than the deflections of RC beams. Nakov et al. [101] saw decreases in total deflections after one year of 18% and 25% for R-SFRC with 0.8% and 1.6% of steel fibers, respectively, relative to RC. In the study by Aslani et al. [95] deflections always decreased with the addition of fibers (~0.5%). Adding only steel fibers was the most effective, followed by mixing steel and polypropylene fibers in equal volumes, whereas adding polypropylene fibers decreased deflections only slightly; nonetheless, all beams experienced deflection stabilization after 240 days. At the same time, results for crack widths are not conclusive for R-SFRC, although generally crack widths tend to be reduced relative to RC.

It should be noted that the majority of the “hybrid-FRC” members (i.e., members with fibers and steel reinforcement) had relatively large reinforcement ratios. It is possible that long-term deflection and crack development will be more critical for members containing fibers and minimum steel reinforcement; therefore, this is something that should be determined in future research. Nonetheless, it can be safely claimed that creep failure of FRC elements in the presence of minimum steel reinforcement is not to be expected.

### 4.2. Serviceability Limit State (SLS) Design of FRC

Plizzari and Serna [26] specify two types of FRC structural applications: “enhancing crack behavior which is particularly important at SLS and also for durability requirements” and “replacing all or part of the conventional reinforcement for structural capacity at Ultimate Limit States (ULS)” [26].

In practice and in cases where cracking is expected under service conditions, hybrid-FRC members are most likely to be used (with fibers and steel reinforcement). Whether fibers are added only for enhancing SLS behavior or whether steel reinforcement is partially replaced, in order to assess deflections and crack widths, the creep behavior of FRC needs to be considered.

Because of the significant uncertainties still related with FRC in general, and its creep behavior in particular, codes tend to apply strict limitations on its properties when it is to be used as a structural material. For example, the *fib* Model Code 2010 [108] requires a minimum “performance class” of “1.0 a” when FRC residual strength is required for equilibrium conditions (complete or even partial reinforcement substitution): the minimum values of *f*_R1k_ and *f*_R3k_ (characteristic residual strengths obtained in the EN 14,651 [112] test at a CMOD of 0.5 and 2.5 mm, respectively) are 1.0, and 0.5 MPa, respectively. Furthermore, *f*_R1k_ must be greater than 0.4·*f*_Lk_, where *f*_Lk_ is the characteristic limit of proportionality as defined by EN 14,651 [112]) and *f*_R3k_ must be greater than 0.5·*f*_R1k_. In terms of ductility, the *fib* Model Code 2010 [108] also requires one of the following conditions to be satisfied:

(5) $$\delta_{u}\geq 20\cdot \delta_{\mathrm{SLS}}$$

(6) $$\delta_{\mathrm{peak}}\geq 5\cdot \delta_{\mathrm{SLS}}$$

where *δ*_u_ is the ultimate displacement of the structure or member, *δ*_peak_ is the displacement at peak load, and *δ*_SLS_ is the displacement under service conditions. In reality, these conditions are very strict, and usually either preclude the use of FRC without reinforcement or require flexural hardening of the structural element [26].

However, besides these general recommendations, there is not much guidance in the *fib* Model Code 2010 [108], or other codes (for example the Spanish EHE-08 [114], in terms of providing specific models or expressions for incorporating creep behavior of FRC into structural analysis and design. Even though certain research in this direction has been conducted and some models have been put forward, currently this area remains the one that is most open to advances.

One of the earliest works in this area was done by Tan et al. [98,99] and continued by Tan and Saha [100]. The authors formulated an adjustment of the ACI 318 Building Code [115] procedure for deflection control making it applicable to SFRC beams, mostly based on own experimental results. The ACI 318 model is based on calculating deflections using an effective moment of inertia (interpolated between the uncracked and fully cracked states). Instantaneous deflections are multiplied by a factor to take into account creep, whereas deflections due to shrinkage are calculated from the induced curvature. In terms of SFRC adjustments, the proposed method provides an expression for the moment of inertia of a cracked section, considering the contribution of fibers through a ratio of the moduli of elasticity of steel fibers and concrete and the equivalent areas of fibers in the compressed and tensile zone. At the level of deflections, the multiplicator for creep effects is adjusted by an empirical formula based only on the volume of steel fibers *V*_f_. These authors also propose obtaining SFRC crack widths by adjusting those calculated for RC members through a linear relation dependent also on the fiber volume *V*_f_. Relatively good agreement of model predictions with results of 10-year beam measurements was found [100]. Since the method was developed on own experimental results, there is room for improvement and generalization of the method by including properties such as tensile creep of FRC.

So far, most work on SLS constitutive modeling and design of SFRC has been undertaken in a sequence of papers by Amin and Gilbert [44,84], Amin et al. [102,103], and Watts et al. [86,87]. The authors started by developing a model for the tension stiffening effect in R-SFRC [102] and then succeeded in applying the model in the calculation of instantaneous crack widths [84] and instantaneous and time-dependent deflections [87,103].

**Figure 10:**
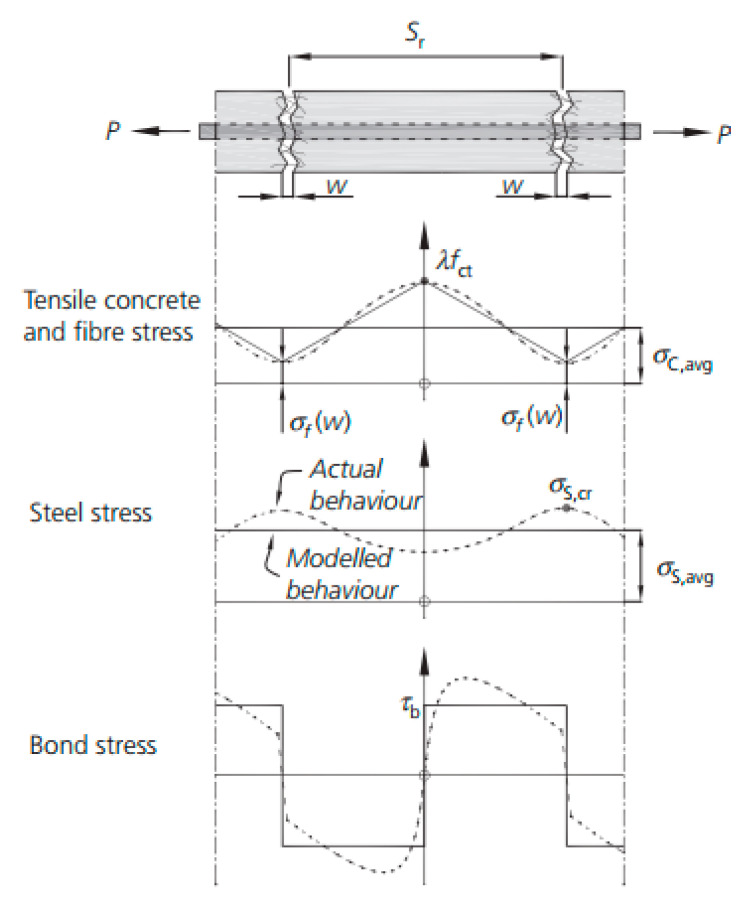
Tension chord including the effect of steel fibers and reinforcing bar (authorized reprint from [102]).

Amin et al. [102] proposed an extension of the so-called tension chord model (TCM) to R-SFRC to model the tension stiffening effect, also explicitly accounting for shrinkage. A general scheme of the model is reproduced in Figure 10, where *λ* is a factor between 0 and 1, *f*_ct_ is the concrete tensile strength, *s*_r_ is the crack spacing, *w* is the crack width, *σ*_f_ is the stress in steel fibers crossing a crack, *σ*_c,avg_ is the average tension-stiffening stress, *σ*_s,cr_ is the steel reinforcement stress in the cracked section, *σ*_s,avg_ is the average stress in steel reinforcement, and *τ*_b_ is the bond stress.

The proposed model allowed these authors to define tension stiffening stresses for minimum and maximum crack spacing scenarios. The results were verified against own experimental results as well as those previously published in literature [102].

Subsequently, Amin et al. [103] applied the TCM model for R-SFRC to calculating instantaneous deflections of members in bending. They built on the model originally proposed by Kenel et al. [116]. In this model, deflections are not calculating by interpolating between deflections calculated for the uncracked and fully cracked states—as is done in the *fib* Model Code 2010 approach [108]—but rather by calculating the deflection of a fully cracked member and reducing it by tension stiffening contributions:

(7) $$a=a_{1}-∆a_{0}-∆a_{1}$$

where *a* is the total deflection, *a*_1_ is the deflection of the member that would be obtained assuming it is fully cracked over its entire length, and Δ*a*_0_ and Δ*a*_1_ are the stiffening effects of uncracked and cracked regions of the member, respectively. The stiffening effects are due to a “curvature offset” Δ*χ* for which the Amin et al. [103] propose a formulation for R-SFRC (based on their TCM model), as well as a method of calculating cracked sectional properties, similar to the method applied by Tan et al. [98]. The method was successfully verified using available experimental data. Watts et al. [87] further applied this model to time-dependent deflections of R-SFRC members. The model is based on Equation (7) and the previous study by Amin et al. [103], expanded by accounting for the increases in curvature over time due to creep (only compressive creep is considered) and shrinkage. A comparison with available test data showed good results. Finally, based on the same TCM model Amin and Gilbert [84] proposed an iterative procedure for instantaneous crack width calculation.

It can be seen that, although current codes still do not incorporate provisions for time-dependent analysis of FRC, progress is being made. The TCM model developed by Amin et al. [102] is a significant way forward. What still remains is the adaptation of the model to PFRC on the tension chord level, as well as the incorporation of time-dependent polymeric fiber properties into long-term deflection predictions. This can potentially be done by modifying sectional properties as is achieved by the effective modulus method and the compressive creep coefficient.

### 4.3. Summary of the Results on Structural Level Testing and Modeling of FRC Creep Behavior

The previous sections have shown that research on the creep behavior of FRC exists on the structural level, both in terms of experimental and theoretical work. However, the body of literature is still insufficient for design and practical purposes and more work is needed until it is mature enough to translate into design codes and wider practical applications by industry.

In terms of experimental research, work should be focused on hybrid-FRC but also on cases with reinforcement ratios close to the minimum; particularly, more experiments are needed on R-PFRC. Furthermore, the current experiments do not provide a clear link between long-term bending or uniaxial tension tests on the material and structural level, i.e., available results on the material level (e.g., crack width creep coefficients) are not applicable to structural tests. In terms of theoretical work, the broadening of current models (whether existing ACI or *fib* Model Code approaches or TCM models) to R-PFRC is still lacking. Current results from numerical simulations are reason for optimism that with more comprehensive testing, formulation, validation, and calibration of theoretical models through numerical parametric studies will be possible.

## 5. Conclusions

In this study, a systematic and critical literature review on the creep behavior of FRC and FRC structural members has been presented. The study covered research on the single fiber level, material level of FRC, and structural level of FRC and hybrid-FRC members, including experimental and theoretical works.

The methodology employed for literature search and filtering is transparently presented and potential biases (only literature in English and journal and conference articles are considered) are discussed. This, notwithstanding the review performed herein, offers several important conclusions from the current state of the art as well as clear recommendations for future research.

On the single fiber level, single fiber creep tests results allow concluding that SFRC might be susceptible to pull-out creep, whilst PFRC is susceptible also to fiber creep provided fibers are subjected to permanent tensile stresses of magnitudes incompatible with the temperature and time-dependent mechanical properties of each type of polymeric fibers. Series Kelvin chain models can describe the creep behavior of both SFRC and PFRC but research is still needed to propose a unified model applicable to both FRC types.

On the FRC material level, there is still a large variety in the ranges of parameter values used in long-term uniaxial tension and bending tests. Future tests should include long-term uniaxial tension or bending tests coupled with shrinkage and compressive creep tests. Then, Kelvin chain models could be applied to the results of such tests to propose a temperature and load-dependent constitutive models that could further be validated using numerical analyses.

Finally, structural-level research has shown promising results in terms of hybrid-FRC performance, both SFRC and PFRC, since long-term increases of deflections and crack widths of hybrid FRC solutions are lower than for traditional RC solutions. Existing theoretical work has forged a way to consider the contribution of fibers in deflection and crack width calculations. Nonetheless, work on SFRC can be expanded to cover PFRC as well. Future research should focus on hybrid-FRC with low reinforcement ratios and PFRC solutions. However, all experiments should be as comprehensive as possible: accompanied by shrinkage, compression, and tensile creep tests so that material-level results could be directly applied to modeling structural behavior. Finally, it should be noted that use of hybrid FRC solutions (both steel and polymeric) with steel reinforcement converts FRC creep to a solvable serviceability-related problem, while at the same time bringing tangible and objective technical and economic benefits.

In conclusion, FRC subjected to creep (especially in the cracked state) is an interesting and dynamic area that has seen significant advances over the past decades but still offers room for improvement, all with the aim of reaching a consensus that will enable code development and industry uptake of structural FRC applications.

## Figures and Tables

**Table 1:** Summary of systematic literature search.

Topic/Group	No. of Studies/References
Total no. of studies	72; [26,28,34,35,36,37,38,39,40,41,42,43,44,45,46,47,48,49,50,51,52,53,54,55,56,57,58,59,60,61,62,63,64,65,66,67,68,69,70,71,72,73,74,75,76,77,78,79,80,81,82,83,84,85,86,87,88,89,90,91,92,93,94,95,96,97,98,99,100,101,102,103]
Fiber level	10; [28,34,35,36,37,38,39,40,41,42]
Concrete level	43; [28,37,43,44,45,46,47,48,49,50,51,52,53,54,55,56,57,58,59,60,61,62,63,64,65,66,67,68,69,70,71,72,73,74,75,76,77,78,79,80,81,82,83]
Structural level	23; [26,44,45,84,85,86,87,88,89,90,91,92,93,94,95,96,97,98,99,100,101,102,103]

**Table 2:** Summary of parameters in long-term uniaxial tension tests.

Ref.	FRC Type	Fiber Aspect Ratio	*V*_f_	Specimens ^1^ (mm)	Pre-Crack Width (mm)	Load Level ^2^	Climate (T & RH) ^3^	Time (Days)
[37]	SFRC	67	0.5%	100/100/500	0.40–0.75	30–85%	–	240
[49]	SFRC	65	1.0%	Ø100/300	0.05; 0.20	30% ^2^	20 °C, 60%	100
[50]	PPFRC	50	1.0%	Ø100/300	0.20	30–45%	20 °C, 60%	180
[79]	PPFRC	50	1.0%	100/100/500	0.50	30–70%	23 °C, 65%	240

^1^ All specimens were notched; ^2^ relative to maximum pre-cracking load; ^3^ temperature and relative humidity.

**Table 3:** Summary of long-term uniaxial tension test results.

Ref.	*f*_max_ (MPa)	*f*_R1_ (MPa)	*f*_R3_ (MPa)	*ϕ*_w_
[37]	3.65	1.76	2.13	0.87–2.10
[49]	6.84; 8.00	6.59; 7.50	6.02; 6.81	0.95–2.10 ^1^ ~0.8–4.0 ^2^
[50]	3.70	1.78	2.05	<1.00 ^3^ ~9 ^4^
[79]	~3.00	~0.80	~1.00	–

^1^ At pre-crack 0.05 mm; ^2^ at pre-crack 0.20 mm; ^3^ at load level 30%; ^4^ at load level 45%.

**Table 4:** Summary of parameters in long-term bending tests.

Ref.	FRC Type	Fiber Aspect Ratio	*V*_f_	Specimens (mm) and Test Type	Pre-Crack Width (mm)	Load Level	Climate (T and RH)	Time (Days)
[47]	PFRC	-	~0.1%	100/100/350 Cantilever	0.75	22–88%	-	Until failure
[48]	PPFRC	50	1.0%	100/100/500 4-point	0.20	30–50%	23 °C; 65%	240
[53]	SFRC	-	0.5	50–150/150/600 4-point	0.50	60%	23 °C	110
[55]	SFRC	45–80	0.5%; 0.9%	150/150/600 4-point	0.50	60%; 80%	-	90
[56]	SFRC	50	1.25%	150/150/600 4-point	0.05–0.50	25–45%	22 °C	180
[57]	SFRC	50	0.50%	150/150/600 4-point	0.2–3.5	64–156% ^1^	16–23 °C; 22–64%	630
[63]	SFRC	44	1.0%; 2.0%	50/50/650 3-point	–	20% ^1^	23 °C	120
[65]	SFRC	65	1.90%	40/80/1200 4-point	n/a ^2^	50% ^2^	-	150
[70]	SFRC; PPFRC	44; 83	0.50%	150/150/600 4-point	0.25; 1.50; 2.50	40–70%	-	90
[71]	SFRC; PPFRC	50–160	0.50%	150/150/600 4-point	0.50	70%	20 °C; 60%	90
[78]	SFRC; PPFRC	40–100	0.40%	150/150/600 4- & 3-point	0.50	50–70%	21 °C	290
[80]	SFRC; PFRC	-	~0.40%	100/100/500 4-point	1.75	50%; 60%	21 °C ^3^	3200
[83]	PFRC	40–100	0.3–0.8%	300/120/2000 3-point	0.2	50%	20–50 °C	90

^1^ Relative to maximum pre-cracking load; ^2^ pre-cracking test stopped after fist cracking; ^3^ aluminium-sealed.

**Table 5:** Summary of parameters in long-term tests on full-scale FRC members.

Ref.	Concrete Type	*ρ* ^1^	*V*_f_	Member (mm)	Load Level ^2^	Time (days)	Main Results
[88]	RC; R-SFRC ^3^	0.45%; 1.25%	0.75%; 1.5%	100/150/3000 beams	0.60	180	Deflections decrease with increasing *V*_f_ up to 0.75%, then remain ~constant
[93]	RC; PPFRC	–	0.40%; 0.80%	Ø600; Ø900 pipes	– ^4^	180	Larger change of vertical displacements for PPFRC but stabilization over time; crack widths continued to increase
[94]	R-PPFRC	0.20%	1.0%	Ø1200 pipes	0.40	120	Deflection increase over 5 days then stabilization; crack widths continued to increase
[95]	RC; R-SFRC; R-PPFRC ^5^; R-MFRC ^6^	0.85%	~0.5% ^7^	400/161/3500 beams	0.30–0.50	240	Deflections decrease in order of RC, R-PPFRC, R-MFRC, R-SFRC
[96]	PPFRC	–	–	Ø600; Ø900 pipes	– ^8^	417	Pre-cracking had a minor effect on the increase of pipe vertical deflection
[97]	RC; R-SFRC; R-PPFRC	0.9%	0.6% ^9^; 0.9% ^10^	250/250/3000 beams	0.50	300	Presence of fibers decreases cracks
[100]	RC; R-SFRC	1.5%	0–2%	100/125/2000 beams	0.50	365	Deflections decrease with increasing *V*_f_
[101]	RC; R-SFRC	0.37%	0.8%; 1.6%	150/280/3000 beams	0.40–0.45	365	Deflections decrease with increasing *V*_f_; no clear trends for crack widths

^1^ Longitudinal reinforcement ratio; ^2^ relative to ultimate load; ^3^ reinforced SFRC member; ^4^ pipes were pre-cracked then buried under load corresponding to cracking load; ^5^ reinforced PPFRC member; ^6^ reinforced mixed FRC (mix of steel and polypropylene fibers); ^7^ only steel, only polypropylene, or steel + polypropylene 1:1 volume ratio; ^8^ pipes were tested at “service load” either uncracked or pre-cracked at “ultimate load; ^9^ steel fiber content; ^10^ polypropylene fiber content.

## References

[1] Di Prisco M., Plizzari G., Vandewalle L. (2009). Fibre reinforced concrete: New design perspectives. Mater. Struct..

[2] (2018). FIB Bulletin 83. Precast Tunnel Segments in Fibre-Reinforced Concrete.

[3] De la Fuente A., Blanco A., Armengou J., Aguado A. (2017). Sustainability based-approach to determine the concrete type and reinforcement configuration of TBM tunnels linings. Case study: Extension line to Barcelona Airport T1. Tunn. Undergr. Space Technol..

[4] De La Fuente A., Casanovas-Rubio M.D.M., Pons O., Armengou J. (2019). Sustainability of Column-Supported RC Slabs: Fiber Reinforcement as an Alternative. J. Constr. Eng. Manag..

[5] WBCSD (2017). The Cement Sustainability Initiative. World Bus. Counc. Sustain. Dev..

[6] Scrivener K.L., Vanderley J.M., Gartner E.M. (2016). Eco-Efficient Cements: Potential, Economically Viable Solutions for a Low-CO2, Cement Based Materials Industry.

[7] Roesler J.R., Altoubat S.A., Lange D.A., Rieder K.A., Ulreich G.R. (2006). Effect of synthetic fibers on structural behavior of concrete slabs-on-ground. ACI Mater. J..

[8] Meda A., Plizzari G.A., Riva P. (2004). Fracture behavior of SFRC slabs on grade. Mater. Struct. Constr..

[9] Alani A.M., Beckett D. (2013). Mechanical properties of a large scale synthetic fibre reinforced concrete ground slab. Constr. Build. Mater..

[10] Meda A., Plizzari G.A. (2004). New design approach for steel fiber-reinforced concrete slabs-on-ground based on fracture mechanics. ACI Struct. J..

[11] Chen S. (2007). Steel fiber concrete slabs on ground: A structural matter. ACI Struct. J..

[12] Caratelli A., Meda A., Rinaldi Z., Romualdi P. (2011). Structural behaviour of precast tunnel segments in fiber reinforced concrete. Tunn. Undergr. Space Technol..

[13] Chiaia B., Fantilli A.P., Vallini P. (2007). Evaluation of minimum reinforcement ratio in FRC members and application to tunnel linings. Mater. Struct. Constr..

[14] Chiaia B., Fantilli A.P., Vallini P. (2009). Combining fiber-reinforced concrete with traditional reinforcement in tunnel linings. Eng. Struct..

[15] De la Fuente A., Pujadas P., Blanco A., Aguado A. (2012). Experiences in Barcelona with the use of fibres in segmental linings. Tunn. Undergr. Space Technol..

[16] Jamshidi Avanaki M., Hoseini A., Vahdani S., de Santos C., de la Fuente A. (2018). Seismic fragility curves for vulnerability assessment of steel fiber reinforced concrete segmental tunnel linings. Tunn. Undergr. Space Technol..

[17] Plizzari G.A., Tiberti G. (2006). Steel fibers as reinforcement for precast tunnel segments. Tunn. Undergr. Space Technol..

[18] Meda A., Rinaldi Z., Caratelli A., Cignitti F. (2016). Experimental investigation on precast tunnel segments under TBM thrust action. Eng. Struct..

[19] De La Fuente A., Escariz R.C., De Figueiredo A.D., Aguado A. (2013). Design of macro-synthetic fibre reinforced concrete pipes. Constr. Build. Mater..

[20] De La Fuente A., Escariz R.C., De Figueiredo A.D., Molins C., Aguado A. (2012). A new design method for steel fibre reinforced concrete pipes. Constr. Build. Mater..

[21] Massicotte B. High performance fibre reinforced concrete for structural applications. Proceedings of the 3rd FRC International Workshop Fibre Reinforced Concrete: From Design to Structural Applications.

[22] Aidarov S., de la Fuente A., Mena F., Ángel S. (2019). Campaña experimental de un forjado de hormigón reforzado con fibras a escala real. Proceedings of the ACE.

[23] Destrée X., Mandl J. Steel fibre only reinforced concrete in free suspended elevated slabs: Case studies, design assisted by testing route, comparison to the latest SFRC standard documents. Proceedings of the International FIB Symposium 2008—Tailor Made Concrete Structures: New Solutions for our Society.

[24] Gossla U. (2005). Development of SFRC Free Suspended Elevated Flat Slabs.

[25] Parmentier B., Van Itterbeeck P., Skowron A. (2014). The Behaviour of SFRC Flat Slabs: The Limelette Full-Scale Experiments to Support Design Model Codes.

[26] Plizzari G., Serna P. (2018). Structural effects of FRC creep. Mater. Struct. Constr..

[27] Ghali A., Favre R., Eldbadry M. (2002). Concrete Structures. Stresses and Deformation.

[28] Gettu R., Zerbino R., Jose S., Serna P., Llano-Tore A., Cavalaro S.H.P. (2017). Factors Influencing Creep of Cracked Fibre Reinforced Concrete: What We Think We Know & What We Do Not Know. Proceedings of the Creep Behaviour in Cracked Sections of Fibre Reinforced Concrete.

[29] Berrocal C.G., Löfgren I., Lundgren K. (2018). The effect of fibres on steel bar corrosion and flexural behaviour of corroded RC beams. Eng. Struct..

[30] Vieira M.D.M. (2018). Assessment of Chloride Corrosion in Steel Fibre Reinforced Cementitious Composites. Ph.D. Thesis.

[31] Bernard E.S. (2020). Changes in long-term performance of fibre reinforced shotcrete due to corrosion and embrittlement. Tunn. Undergr. Space Technol..

[32] Science Direct Scopus. https://www.scopus.com/home.uri.

[33] Clarivate Analytics Web of Science. www.webofknowledge.com.

[34] Liu X., Huang Y., Deng C., Wang X., Tong W., Liu Y., Huang J., Yang Q., Liao X., Li G. (2009). Study on the Creep Behavior of Polypropylene. Polym. Eng. Sci..

[35] Vrijdaghs R., di Prisco M., Vandewalle L., Serna P., Llano-Tore A., Cavalaro S.H.P. (2017). Creep Deformations of Structural Polymeric Macrofibers. Proceedings of the Creep Behaviour in Cracked Sections of Fibre Reinforced Concrete.

[36] Vrijdaghs R., di Prisco M., Vandewalle L. (2017). Short-term and creep pull-out behavior of polypropylene macrofibers at varying embedded lengths and angles from a concrete matrix. Constr. Build. Mater..

[37] Nieuwoudt P.D., Babafemi A.J., Boshoff W.P. (2017). The response of cracked steel fibre reinforced concrete under various sustained stress levels on both the macro and single fibre level. Constr. Build. Mater..

[38] Babafemi A.J., du Plessis A., Boshoff W.P. (2018). Pull-out creep mechanism of synthetic macro fibres under a sustained load. Constr. Build. Mater..

[39] Fouda I.M., El-Farahaty K.A., Seisa E.A. (2008). Interferometric Study of Creep Deformation and Some Structural Properties of Polypropylene Fiber at Three Different Temperatures. J. Appl. Polym. Sci..

[40] Hadley D.W., Ward I.M. (1965). Non-linear creep and recovery behaviour of polypropylene fibres. J. Mech. Physcs Solids.

[41] Sabuncuoglu B., Acar M., Silberschmidt V.V. (2011). Analysis of creep behavior of polypropylene fibers. Appl. Mech. Mater..

[42] Takaku A. (1980). Effect of temperature on creep fracture of polypropylene fibers. J. Appl. Polym. Sci..

[43] Pujadas P., Blanco A., Cavalaro S.H.P., De La Fuente A., Aguado A., Serna P., Llano-Tore A., Cavalaro S.H.P. (2017). Flexural Post-cracking Creep Behaviour of Macro-synthetic and Steel Fiber Reinforced Concrete. Proceedings of the Creep Behaviour in Cracked Sections of Fibre Reinforced Concrete.

[44] Amin A., Gilbert R.I. (2019). Steel fiber-reinforced concrete beams—Part I: Material characterization and in-service behavior. ACI Struct. J..

[45] Amin A., Ian Gilbert R. (2019). Steel fiber-reinforced concrete beams—Part II: Strength, ductility, and design. ACI Struct. J..

[46] Kusterle W. Viscous material behavior of solids-creep of polymer fiber reinforced concrete. Proceedings of the 5th Central European Congress on Concrete Engineering.

[47] Kurtz S., Balaguru P. (2000). Postcrack creep of polymeric fiber-reinforced concrete in flexure. Cem. Concr. Res..

[48] Babafemi A.J., Boshoff W.P. (2016). Testing and modelling the creep of cracked macro-synthetic fibre reinforced concrete (MSFRC) under flexural loading. Mater. Struct. Constr..

[49] Zhao G., di Prisco M., Vandewalle L. (2015). Experimental investigation on uniaxial tensile creep behavior of cracked steel fiber reinforced concrete. Mater. Struct. Constr..

[50] Vrijdaghs R., di Prisco M., Vandewalle L. (2018). Uniaxial tensile creep of a cracked polypropylene fiber reinforced concrete. Mater. Struct..

[51] Noushini A., Castel A., Gilbert R.I. (2019). Creep and shrinkage of synthetic fibre-reinforced geopolymer concrete. Mag. Concr. Res..

[52] Nakov D., Markovski G., Arangjelovski T., Mark P. (2018). Experimental and analytical analysis of creep of steel fibre reinforced concrete. Period. Polytech. Civ. Eng..

[53] Zerbino R., Giaccio G., Monetti D., Torrijos M., Serna P., Llano-Tore A., Cavalaro S.H.P. (2017). Effect of Beam Width on the Creep Behaviour of Cracked Fibre Reinforced Concrete. Proceedings of the Creep Behaviour in Cracked Sections of Fibre Reinforced Concrete.

[54] Pešić N., Živanović S., Garcia R., Papastergiou P. (2016). Mechanical properties of concrete reinforced with recycled HDPE plastic fibres. Constr. Build. Mater..

[55] García-Taengua E., Arango S., Martí-Vargas J.R., Serna P. (2014). Flexural creep of steel fiber reinforced concrete in the cracked state. Constr. Build. Mater..

[56] Monetti D.H., Llano-Torre A., Torrijos M.C., Giaccio G., Zerbino R., Martí-Vargas J.R., Serna P. (2019). Long-term behavior of cracked fiber reinforced concrete under service conditions. Constr. Build. Mater..

[57] Zerbino R.L., Barragán B.E. (2012). Long-term behavior of cracked steel fiber-reinforced concrete beams under sustained loading. ACI Mater. J..

[58] Afroughsabet V., Teng S. (2020). Experiments on drying shrinkage and creep of high performance hybrid-fiber-reinforced concrete. Cem. Concr. Compos..

[59] Babafemi A.J., Boshoff W.P. (2013). Time-dependent behaviour of pre-cracked polypropylene fibre reinforced concrete (PFRC) under sustained loading. Res. Appl. Struct. Eng. Mech. Comput..

[60] Błyszko J. (2017). Comparative Analysis of Creep in Standard and Fibre Reinforced Concretes under different Load Conditions. Procedia Eng..

[61] Teixeira Buttignol T.E., Colombo M., di Prisco M. (2016). Long-term aging effects on tensile characterization of steel fibre reinforced concrete. Struct. Concr..

[62] Chern J.C., Young C.H. (1989). Compressive creep and shrinkage of steel fibre reinforced concrete. Int. J. Cem. Compos. Light. Concr..

[63] Chern J.C., Young C.H. (1992). Pickett effect and creep in flexure of steel-fiber reinforced concrete. J. Chinese Inst. Eng. Trans. Chinese Inst. Eng..

[64] Babafemi A.J., Boshoff W.P., Serna P., Llano-Tore A., Cavalaro S.H.P. (2017). Macro-Synthetic Fibre Reinforced Concrete: Creep and Creep Mechanisms. Proceedings of the Creep Behaviour in Cracked Sections of Fibre Reinforced Concrete.

[65] Galeote E., Blanco A., de la Fuente A., Cavalaro S.H.P. (2017). Creep Behaviour of Cracked High Performance Fibre Reinforced Concrete Beams Under Flexural Load. Proceedings of the Creep Behaviour in Cracked Sections of Fibre Reinforced Concrete.

[66] Kohoutkova A., Vodička J., Kristek V. (2015). Creep and Shrinkage of Fibre-Reinforced Concrete and a Guide for Modeling. Proceedings of the CONCREEP 10.

[67] Marangon E., Toledo Filho R.D., Fairbairn E.M.R. (2012). Basic Creep under Compression and Direct Tension Loads of Self-compacting-steel Fibers Reinforced Concrete. High Performance Fiber Reinforced Cement Composites 6.

[68] Llano-Torre A., García-Taengua E., Martí-Vargas J.R., Serna P. Compilation and study of a database of tests and results on flexural creep behavior of fibre reinforced concrete specimens. Proceedings of the FIB Symposium Concrete Innovation and Design.

[69] Nürnbergerová T., Babál B. (1992). Long-term behaviour of plain and steel fibre reinforced concrete rings. Mater. Struct..

[70] Pujadas P., Blanco A., Cavalaro S., de la Fuente A., Aguado A. (2017). The need to consider flexural post-cracking creep behavior of macro-synthetic fiber reinforced concrete. Constr. Build. Mater..

[71] Serna P., Martí-Vargas J.R., Bossio M.E., Zerbino R. (2016). Creep and residual properties of cracked macro-synthetic fibre reinforced concretes. Mag. Concr. Res..

[72] Sturm A.B., Visintin P., Oehlers D.J., Seracino R. (2018). Time-Dependent Tension-Stiffening Mechanics of Fiber-Reinforced and Ultra-High-Performance Fiber-Reinforced Concrete. J. Struct. Eng..

[73] Sprince A., Korjakins A., Pakrastinsh L. (2013). Time-Dependent Behavior of High Performance Fiber-Reinforced Concrete.

[74] Vrijdaghs R., Verstrynge E., Vandewalle L., di Prisco M. (2018). A two-phased and multi-scale finite element analysis of the tensile creep behavior of polypropylene fiber reinforced concrete. Conference on Computational Modelling of ConcreteÂ and Concrete Structures, EURO-C 2018.

[75] Buratti N., Mazzotti C., Serna P., Llano-Tore A., Cavalaro S.H.P. (2017). Creep Testing Methodologies and Results Interpretation. Proceedings of the Creep Behaviour in Cracked Sections of Fibre Reinforced Concrete.

[76] Vrijdaghs R., di Prisco M., Vandewalle L. (2020). Creep of polymeric fiber reinforced concrete: A numerical model with discrete fiber treatment. Comput. Struct..

[77] Vrijdaghs R., di Prisco M., Vandewalle L. (2017). A Numerical Model for the Creep of Fiber Reinforced Concrete. Proceedings of the High Tech Concrete: Where Technology and Engineering Meet.

[78] Zerbino R., Monetti D.H., Giaccio G. (2016). Creep behaviour of cracked steel and macro-synthetic fibre reinforced concrete. Mater. Struct. Constr..

[79] Babafemi A.J., Boshoff W.P. (2015). Tensile creep of macro-synthetic fibre reinforced concrete (MSFRC) under uni-axial tensile loading. Cem. Concr. Compos..

[80] Kusterle W., Serna P., Llano-Tore A., Cavalaro S.H.P. (2017). Flexural Creep Tests on Beams—8 Years of Experience with Steel and Synthetic Fibres. Proceedings of the Creep Behaviour in Cracked Sections of Fibre Reinforced Concrete.

[81] Van Bergen S., Pouillon S., Vitt G., Serna P., Llano-Tore A., Cavalaro S.H.P. (2017). Experiences from 14 Years of Creep Testing of Steel and Polymer Fiber Reinforced Concrete. Proceedings of the Creep Behaviour in Cracked Sections of Fibre Reinforced Concrete.

[82] Arango S.E., Serna P., Martí-Vargas J.R., García-Taengua E. (2012). A Test Method to Characterize Flexural Creep Behaviour of Pre-cracked FRC Specimens. Exp. Mech..

[83] Buratti N., Mazzotti C. (2015). Experimental tests on the effect of temperature on the long-term behaviour of macrosynthetic Fibre Reinforced Concretes. Constr. Build. Mater..

[84] Amin A., Gilbert R.I. (2018). Instantaneous crack width calculation for steel fiber-reinforced concrete flexural members. ACI Struct. J..

[85] Raymond A., Gilbert I., Bernard C.E.S. Time-dependent Analysis of Macro-synthetic FRC Sections with Bar Reinforcement. Proceedings of the ITA WTC 2015 Congress and 41st General Assembly.

[86] Watts M.J., Amin A., Gilbert R.I., Kaufmann W. (2020). Behavior of fiber reinforced concrete members under sustained axial/flexural load. Struct. Concr..

[87] Watts M.J., Amin A., Gilbert R.I., Kaufmann W., Minelli F. (2020). Simplified prediction of the time dependent deflection of SFRC flexural members. Mater. Struct. Constr..

[88] Ashour S.A., Mahmood K., Wafa F.F. (1999). Long-term deflection of high-strength fiber reinforced concrete beams. Struct. Eng. Mech..

[89] Ezeldin A.S., Shiah T.W. (1995). Analytical immediate and long-term deflections of fiber-reinforced concrete beams. J. Struct. Eng..

[90] Habel K., Denarié E., Brühwiler E. (2006). Time dependent behavior of elements combining ultra-high performance fiber reinforced concretes (UHPFRC) and reinforced concrete. Mater. Struct. Constr..

[91] Kaminski M., Bywalski C. Influence of creep deformations on value of long term deflections of steel fiber-reinforced concrete beams. Proceedings of the 8th International Conference on Creep, Shrinkage and Durability of Concrete and Concrete Structures.

[92] Neutov S., Sydorchuk M., Surianinov M. (2019). Experimental studies of reinforced concrete and fiber-reinforced concrete beams with short-term and long-term loads. Mater. Sci. Forum.

[93] Park Y., Abolmaali A., Attiogbe E., Lee S.H. (2014). Time-dependent behavior of synthetic fiber-reinforced concrete pipes under long-term sustained loading. Transp. Res. Rec..

[94] Al Rikabi F.T., Sargand S.M., Khoury I., Kurdziel J. (2020). A new test method for evaluating the long-term performance of fiber-reinforced concrete pipes. Adv. Struct. Eng..

[95] Aslani F., Nejadi S., Samali B. (2014). Long-term flexural cracking control of reinforced self-compacting concrete one way slabs with and without fibres. Comput. Concr..

[96] Attiogbe E., Abolmaali A., Park Y. Polypropylene fiber-reinforced concrete pipes under sustained loading. Proceedings of the 9th RILEM International Symposium on Fiber Reinforced Concrete—BEFIB 2016.

[97] Vasanelli E., Micelli F., Aiello M.A., Plizzari G. (2013). Long term behavior of FRC flexural beams under sustained load. Eng. Struct..

[98] Tan K.H., Paramasivam P., Tan K.C. (1994). Instantaneous and long-term deflections of steel fiber reinforced concrete beams. ACI Struct. J..

[99] Tan K.H., Paramasivam P., Tan K.C. (1994). Creep and shrinkage deflections of RC beams with steel fibers. J. Mater. Civ. Eng..

[100] Tan K.H., Saha M.K. (2005). Ten-year study on steel fiber-reinforced concrete beams under sustained loads. ACI Struct. J..

[101] Nakov D., Markovski G., Arangjelovski T., Mark P. (2017). Creeping effect of SFRC elements under specific type of long term loading. RILEM Bookseries.

[102] Amin A., Foster S.J., Watts M. (2016). Modelling the tension stiffening effect in SFR-RC. Mag. Concr. Res..

[103] Amin A., Foster S.J., Kaufmann W. (2017). Instantaneous deflection calculation for steel fibre reinforced concrete one way members. Eng. Struct..

[104] Banthia N., Gupta R. (2006). Influence of polypropylene fiber geometry on plastic shrinkage cracking in concrete. Cem. Concr. Res..

[105] ACI Committee 544 (2002). Report on Fiber Reinforced Concrete.

[106] Bažant Z.P., Baweja S. (1995). Creep and shrinkage prediction model for analysis and design of concrete structures—Model B3. Mater. Struct..

[107] Bažant Z.P., Li G.H. NU-ITI Database on Concrete Creep and Shrinkage. http://iti.northwestern.edu/publications/bazant/index.html.

[108] FIB (2013). fib Model Code for Concrete Structures 2010.

[109] Bažant Z.P., Hubler M.H., Wendner R. (2015). RILEM draft recommendation: TC-242-MDC multi-decade creep and shrinkage of concrete: Material model and structural analysis. Mater. Struct..

[110] Gilbert R.I., Ranzi G. (2011). Time-Dependent Behaviour of Conrete Structures.

[111] Neville A.M. (1995). Properties of Concrete.

[112] (2005). EN 14651 Test Method for Metallic Fibred Concrete—Measuring the Flexural Tensile Strength (Limit of Proportionality (LOP), Residual).

[113] (2004). Eurocode 2: Design of Concrete Structures—Part 1-1: General Rules and Rules for Buildings.

[114] EHE (2008). Instrucción de Hormigón Estructural (EHE-08).

[115] ACI 318-14 (2014). Building Code Requirements for Structural Concrete (ACI 318-14) and Commentary.

[116] Kenel A., Nellen P., Frank A., Marti P. (2005). Reinforcing steel strains measured by Bragg grating sensors. J. Mater. Civ. Eng..

